# The wellbeing of women and men with and without disabilities: evidence from cross-sectional national surveys in 27 low- and middle-income countries

**DOI:** 10.1007/s11136-022-03268-y

**Published:** 2022-10-22

**Authors:** Eric Emerson, Gwynnyth Llewellyn

**Affiliations:** 1 Centre for Disability Research, Faculty of Health & Medicine, Lancaster University, Lancaster, LA1 4YW UK; 2 Centre for Disability Research and Policy, Faculty of Medicine and Health, University of Sydney, Sydney, NSW 2141 Australia; 3 Centre of Research Excellence in Disability and Health, University of Sydney, Sydney, NSW 2141 Australia

**Keywords:** Disability, Personal wellbeing, Discrimination, Violence

## Abstract

**Background:**

Little is known about disability-related inequities in personal wellbeing (PWB) in low- and middle-income countries (LMICs).

**Method:**

Secondary analysis of data collected in Round 6 of UNICEF’s Multiple Indicator Cluster Surveys (MICS) undertaken in 27 LMICs (*n* = 296,693 women, 66,557 men). Data were aggregated across countries by mixed effects multi-level modelling and meta-analysis.

**Results:**

Women and men with disabilities were less likely than their non-disabled peers to report being happy and to be satisfied with their life. These differences were evident in all countries for women and in 18 of 22 countries for men. Aggregated data indicated that: (1) women with disabilities were 14–15% less likely to be happy and 17% less likely to be satisfied with their lives; (2) men with disabilities were 15–17% less likely to be happy and 17–19% less likely to be satisfied with their lives; (3) disability-related inequalities in personal wellbeing were reduced by 22–26% for women and 11–22% for men by adjusting for differences in living conditions.

**Conclusions:**

Future releases of MICS data could prove a valuable resource in monitoring country-level progress to realising Sustainable Development Goal 3 and the extent to which progress is equitable between women and men and between people with/without disabilities. Results also suggest that a significant proportion of the disability-related inequities in wellbeing may be accounted for by modifiable differences in their living conditions and life experiences.

**Supplementary Information:**

The online version contains supplementary material available at 10.1007/s11136-022-03268-y.

## Plain English Summary

Very little is known about the personal wellbeing of women and men with disability living in low- and middle-income countries. We attempted to redress this issue by looking at the personal wellbeing of women and men with and without disability in 27 countries. We found that both women and men with disabilities had lower personal wellbeing than their non-disabled peers. This difference was, in part, due to their increased risk of experiencing discrimination and violence.

## Introduction

Sustainable Development Goal 3 (SDG3) calls for ensuring healthy lives and promoting well-being for all, including the estimated 1 billion people in the world who have a disability [1, 2]. Monitoring progress toward the attainment of SDG3 requires the collection of nationally robust data on wellbeing over time that can be disaggregated by key characteristics such as disability.

International organisations and high-income countries are making increasing use of measures of personal wellbeing (PWB) to monitor social progress [3–5]. PWB has been defined as ‘good mental states, including all of the various evaluations, positive and negative, that people make of their lives and the affective reactions of people to their experiences’ [6]. It is recognised as involving four distinct components: life satisfaction (‘cognitive’/ ‘evaluative’ wellbeing), positive affect (e.g., happiness), negative affect (e.g., anxiety), and eudemonic wellbeing (a sense of worth/purpose/meaning in life) [7]. PWB is consistent with the WHO’s definition of health as a state of complete physical, mental and social well-being and not merely the absence of disease or infirmity.

Population-based studies undertaken in high-income countries have consistently reported that adults with disabilities have lower mean levels of PWB than adults without disabilities [8–13]. The lower PWB of people with disabilities is likely to reflect a greater discrepancy between their evaluation of their current circumstances and their aspirations (the latter being influenced by hedonic adaptation and social comparison) than is the case for non-disabled people (cf., [14]. Indeed, the evidence from cross-sectional studies suggests that people with disabilities are more likely than their peers to be exposed to life conditions associated with poorer PWB such as social and material deprivation, social isolation and loneliness, lack of employment and exposure to a range of potentially adverse life events (e.g., violence and discrimination) [1, 2]. It should be noted, however, that the difference in PWB between people with and without disabilities is often considered less than would be expected (often termed the ‘disability paradox’) [15].

It should be noted, however, that the vast majority of the available evidence on the association between disability and PWB, especially that generated from nationally representative studies, has been generated in high income countries. Very little robust data is available on disability-related inequities in PWB in low- and middle-income countries (LMICs; where 84% of the world’s population and over 80% of people with disabilities live). Only two published studies have used sampling frames that could generate nationally representative estimates of the strength of association between disability and PWB in LMICs. Both were undertaken on adults aged 50 + . First, Rahman et al. [16] reported that in six LMICs (Ghana, China, India, Russia, South Africa and Mexico) older adults with disabilities reported significantly lower life satisfaction than those without disabilities. Second, Gomez-Olive et al. [17] reported that in pooled analyses across eight LMICs (Ghana, Kenya, Tanzania, South Africa, Vietnam, India, Indonesia and Bangladesh) there was a significant negative correlation between disability scores and life satisfaction. In addition, a recent UNICEF report highlighted the lower levels of happiness and life satisfaction among adolescents (age 15–17) in a number of LMICs [18].

Given that most people, including those with disabilities, live in LMICs, the paucity of nationally representative data on the wellbeing of people with/without disabilities in LMICs is highly problematic for attempts to generate global and regional estimates of disability-related inequity in PWB and in monitoring trends in absolute and relative PWB over time. As such, disability related inequalities can remain hidden, misunderstood, or neglected in government policy aiming to reduce inequalities as required by SDG3. The aims of the present study were to: (1) estimate the prevalence of PWB among women and men with/without disabilities in a range of LMICs; and (2) to investigate the extent to which any observed differences may have been accounted for by differences in the living conditions and life experiences between women and men with/without disabilities.

## Method

We undertook secondary analysis of nationally representative data collected in Round 6 (2017-) of UNICEF’s Multiple Indicator Cluster Surveys (MICS) [19]. The MICS survey programme, initiated in the mid-1990s, is the primary data source used by UN agencies to measure progress toward the attainment of the Millennium Development Goals and now the Sustainable Development Goals that apply to the wellbeing of women and children [19, 20]. While administered by country-level agencies (often the national statistical office), technical support is provided by UNICEF. In Round 6 MICS, indicators related to areas such as: child survival; reproductive and maternal health; child health, nutrition and development; childhood education and in-home support for learning; protection of children and women from violence and exploitation; livening in a safe and clean environment; and having an equitable chance in life.

Following approval by UNICEF, MICS data were downloaded from http://mics.unicef.org/. Data were extracted from the MICS household module, the module applied to all women aged 15–49 years in the household and the optional module applied to all men aged 15–49 years in 50% of households [20]. All countries used cluster sampling methods to derive samples representative of the national population. Specific details of the sampling procedure and ethical review procedures used in each country are available at http://mics.unicef.org/. At the end of the download period (1 February 2022), surveys containing data on disability status and PWB were available for 27 countries (Table 1). Women’s data were collected in all countries. The collection of optional men’s data was undertaken in 22 of the 27 countries.

**Table 1:** Country-level survey data for 28 middle- and low-income countries

				Women					Men				
	Year of survey	pcGNI 2018	HDI 2018	Response rate (%)	Sample size^a^	Prevalence of disability with 95% CI	Prevalence of happiness with 95% CI	Prevalence of high subjective wellbeing with 95% CI	Response rate	Sample size^a^	Prevalence of disability with 95% CI	Prevalence of happiness with 95% CI	Prevalence of high subjective wellbeing with 95% CI
Overall prevalence						14.6% (14.1–15.2)	72.2% (71.4–72.9)	55.5% (54.8–56.1)			10.2% (8.8–11.8)	69.5% (67.4–71.5)	48.0% (45.5–50.6)
Upper-middle income													
Costa Rica	2018	$11,590	0.794	82.5	6902	25.4% (23.7–27.2)	87.3% (85.9–88.6)	89.0% (87.5–90.4)	Men’s data not collected				
Montenegro	2018/19	$8430	0.816	54.9	2753	9.6% (5.4–16.2)	93.8% (87.2–97.1)	91.2% (86.1–94.6)	39.3%	757	6.3% (4.0–9.7)	95.7% (92.9–97.4)	89.5% (84.8–92.8)
Belarus	2019	$5700	0.823	93.4	5270	6.8% (5.6–8.0)	91.4% (90.2–92.5)	84.7% (83.1–86.3)	84.5%	2,171	5.2% (3.9–6.9)	85.4% (83.1–87.4)	77.7% (74.9–80.3)
North Macedonia	2018/19	$5470	0.759	83.8	4245	18.2% (11.0–28.3)	85.5% (78.3–90.6)	76.9% (65.3–85.6)	Men’s data not collected				
Tuvalu	2019/20	$5430	n/a	94.6	762	16.9% (15.7–18.2)	95.6% (95.5–95.7)	75.6% (73.4–77.3)	94.6%	271	16.9% (14.6–19.6)	91.9% (91.5–92.3)	59.3% (55.2–63.3)
Suriname	2018	$5210	0.724	74.0	6261	16.6% (15.6–17.4)	82.9% (82.4–84.2)	85.0% (84.0–85.9)	63.4%	2,449	8.7% (8.3–10.6)	83.5% (83.0–85.9)	69.0% (68.2–71.8)
Iraq	2018	$5040	0.689	98.2	26,752	17.0% (16.1–18.0)	66.7% (65.2–68.3)	55.3% (53.4–57.2)	Men’s data not collected				
Georgia	2018	$4450	0.786	75.4	6461	20.1% (18.7–21.6)	87.4% (86.1–88.6)	69.4% (67.6–71.1)	57.2%	2,476	12.1% (10.2–14.6)	84.1% (81.7–86.2)	62.0% (59.1–64.8)
Tonga	2019	$4300	0.717	90.3	2493	9.2% (7.1–11.9)	93.1% (91.6–94.3)	92.5% (91.1–93.7)	83.3%	1,048	7.4% (5.2–10.4)	95.8% (93.8–97.2)	92.2% (89.3–94.3)
Lower-middle income													
Palestine	2019/20	$4190	0.708	92.9	9794	9.5% (8.7–10.5)	80.5% (79.3–81.9)	68.8% (66.8–70.7)	Men’s data not collected				
Samoa	2019/20	$4020	0.704	88.9	3695	7.5% (6.4–8.9)	92.4% (91.1–93.5)	90.1% (88.0–91.8)	79.9%	1,047	7.6% (5.7–10.0)	94.2% (92.0–95.8)	90.7% (88.0–92.8)
Mongolia	2018	$3660	0.735	90.4	9872	21.3% (19.8–22.8)	84.4% (82.9–85.2)	68.9% (67.0–70.8)	79.8%	4,042	16.9% (14.9–19.1)	86.0% (84.1–87.7)	64.1% (61.5–66.7)
Kiribati	2018/19	$3140	0.623	96.7	3806	21.7% (19.8–23.9)	81.5% (78.7–84.0)	76.1% (73.2–78.9)	95.4%	1,866	19.1% (16.7–21.8)	73.9% (69.3–77.5)	72.4% (68.5–76.0)
Ghana	2017/18	$2130	0.596	97.8	12,528	21.9% (20.4–23.4)	71.8% (70.2–73.3)	47.4% (45.6–49.2)	96.7%	4,309	15.6% (13.8–17.6)	73.2% (70.0–76.2)	40.3% (37.7–43.0)
Sao Tome & Principe	2019	$1870	0.624	94.8	2638	25.5% (23.2–28.0)	75.2% (72.8–77.4)	59.1% (56.4–61.7)	87.8%	1,160	11.4% (9.5–13.7)	82.2% (79.4–84.7)	53.0% (49.1–56.9)
Zimbabwe	2018/19	$1790	0.563	92.8	8888	12.6% (11.9–13.3)	57.7% (56.2–59.2)	34.8% (33.4–36.1)	87.6%	3,440	9.7% (8.6–10.9)	62.1% (59.8–64.3)	27.2% (25.4–29.3)
Bangladesh	2019	$1750	0.614	93.1	57,699	11.7% (11.4–12.1)	83.3% (82.9–83.7)	48.3% (47.8–48.9)	Men’s data not collected				
Lesotho	2018	$1390	0.518	86.0	5630	13.2% (12.1–14.5)	64.3% (62.1–66.5)	45.3% (43.3–47.3)	80.6%	2,425	10.7% (9.3–12.4)	71.2% (68.0–74.2)	38.4% (35.2–41.6)
Nepal	2019	$970	0.579	98.3	13,320	8.8% (8.1–9.7)	61.0% (59.2–62.6)	70.7% (68.9–72.3)	97.9%	4,856	6.4% (5.7–7.1)	63.0% (61.3–64.0)	66.6% (65.2–70.9)
Low-income													
Guinea-Bissau	2018/19	$750	0.461	97.6	9597	8.2% (7.0–9.5)	91.1% (89.8–92.2)	74.2% (71.9–76.3)	92.4%	2,391	2.9% (2.1–3.9)	93.2% (90.8–95.0)	41.1% (36.6–45.8)
The Gambia	2018	$710	0.466	94.0	11,790	9.5% (8.7–10.3)	72.6% (71.3–73.9)	48.2% (46.2–50.1)	85.3%	3,745	9.2% (7.9–10.6)	70.3% (67.5–73.0)	48.8% (46.0–51.6)
Chad	2019	$680	0.397	98.6	19,266	18.5% (17.2–19.8)	56.2% (54.3–57.8)	47.4% (45.7–49.2)	97.6%	5,674	8.1% (7.0–9.4)	61.3% (58.5–64.0)	35.2% (32.2–38.2)
Togo	2017	$660	0.513	93.9	6411	22.5% (20.7–24.4)	58.8% (56.9–60.6)	42.5% (40.2–44.9)	91.4%	1,960	12.3% (10.5–14.5)	53.7% (50.0–57.3)	34.4% (30.7–38.3)
DR Congo	2017/18	$490	0.459	99.6	18,978	14.1% (12.6–15.7)	49.0% (45.2–52.9)	42.7% (39.2–46.2)	99.2%	5,191	8.6% (7.2–10.4)	47.5% (43.1–52.0)	34.6% (30.4–39.1)
Sierra Leone	2017	$490	0.438	98.9	15,649	6.2% (5.8–6.5)	72.9% (71.7–73.1)	47.1% (46.3–47.8)	98.1%	6,379	3.3% (2.8–3.6)	73.4% (72.7–74.8)	52.5% (51.8–54.4)
Central African Republic	2018/19	$490	0.381	92.2	8122	33.2% (31.6–35.0)	45.5% (43.3–47.7)	43.0% (40.7–45.4)	86.9%	3,362	12.4% (10.8–14.2)	42.8% (38.7–47.0)	41.6% (37.5–45.9)
Malawi	2019/20	$350	0.485	94.6	21,124	12.8% (12.1–13.6)	61.2% (60.0–62.4)	41.3% (40.0–42.6)	86.5%	5,611	11.7% (9.6–12.2)	55.3% (52.9–57.7)	34.0% (31.7–36.5)

*pcGNI* per capita Gross National Income, *HDI* Human Development Index, *CI* confidence interval

^a^Sample sizes are unweighted and for respondents aged 18–49 with valid disability data

## Disabilities

In Round 6 of MICS the Washington Group Short Set of Questions on Functioning (WGSS) were introduced to identify adults with disability aged 18–49 years. The module was developed by the Washington Group on Disability Statistics, a City Group created under the auspices of the United Nations Statistical Commission, to provide a simple measure of disability/functioning that could be used in national censuses and major national and international health and social surveys [21]. The module is based on informant report of difficulties in six functional domains (seeing, hearing, walking, remembering/concentrating, self-care, communicating). Four response options were available for each domain (‘*no difficulty*’, ‘*yes—some difficulty*’, ‘*yes—a lot of difficulty*’, ‘*cannot do at all*’). Disability is defined by as having ‘a lot of difficulty’ or ‘cannot do at all’ in one or more domain. We used this criterion for identifying respondents with ‘more severe’ disability. While used extensively (see https://www.washingtongroup-disability.com/resources/published-materials/), concern has been expressed about the under identification of disability by the WGSS [22–24]. As a result, we also used the procedure outlined by Bourke and colleagues to identify respondents with ‘less severe’ disability if they were not identified as having ‘more severe’ disability, but did report ‘some difficulty’ in two or more functional domains. Disability data were missing for < 0.1% of respondents.

## Wellbeing

Wellbeing was assessed by responses to two questions:

1. ‘I would like to ask you some simple questions on happiness and satisfaction. First, taking all things together, would you say you are very happy, somewhat happy, neither happy nor unhappy, somewhat unhappy or very unhappy?’ As recommended by UNICEF, we recoded responses to this item into a binary variable (happiness); somewhat unhappy/very unhappy/neither happy nor unhappy vs. very happy/somewhat happy (MICS Indicator EQ10b).
2. ‘Now, look at this ladder with steps numbered from 0 at the bottom to 10 at the top. Suppose we say that the top of the ladder represents the best possible life for you and the bottom of the ladder represents the worst possible life for you. On which step of the ladder do you feel you stand at this time?’ (A version of Cantril's ladder of life satisfaction scale [25]). Given MICS does not include a binary indicator of life satisfaction, we followed the same procedure as used for happiness by recoding responses to this variable into a binary variable split at the scale midpoint (positive life satisfaction; 6–10 vs. 0–5).

Happiness data were missing for 0.1% of respondents and life satisfaction data were missing for 0.2% of respondents for who valid disability data were available.

## Potential predictors of wellbeing

### Country characteristics

Two characteristics of countries were extracted:

1. National wealth: We used World Bank 2018 country income classifications as upper middle income, lower middle income and low income [26]. These classifications are based on per capita Gross National Income (pcGNI; expressed as current US$ rates) using the World Bank’s Atlas Method. We also downloaded 2018 Atlas Method pcGNI from the World Bank website between April and December 2021 [27, 28]. These data were available for all countries.
2. Human Development Index (HDI): The composite HDI integrates three dimensions of human development: life expectancy at birth; mean years of schooling and expected years of schooling; and gross national income per capita [29, 30]. The HDI is the geometric mean of normalized indices for each of the three dimensions. HDI data for 2018 were taken from the 2019 Human Development Report [31]. HDI data were available for all but one country.

### Age

Age was recoded into 18–19 years and then 5-year age groups from 20 to 49 years. Age was treated as a categorical variable in all analyses. Age data were complete for all respondents.

### Marital status

Marital status was recorded as: (1) currently married/in union; (2) formerly married/in union; (3) never married/in union. Data were missing for 0.1% of respondents for who valid disability data were available.

### Household wealth

MICS data includes a within-country wealth index for each household. Each household is assigned a wealth score based on the assets owned by that household weighted by factors scores. The wealth index is assumed to capture underlying long-term wealth through information on the household assets [32–35]. Data were complete for all respondents.

### Highest level of education

The highest level of education received by each woman in the household was recorded using country-specific categories. We recoded these data into a three-category measure: (1) no education; (2) primary education; (3) receipt of secondary or higher-level education. Data were missing for < 0.1% of respondents for who valid disability data were available.

### Urban/rural location

Data were released with a within-country defined binary indicator of urban/rural location for each household. Data were missing for one country (Belarus). No data were missing in other countries.

### Violence & discrimination

Two sets of questions addressed exposure to violence in the previous year with the following preamble: ‘Now I would like to ask you some questions about crimes in which you personally were the victim. Let me assure you again that your answers are completely confidential and will not be told to anyone.’

1. ‘In the last 3 years, that is since (month of interview) (year of interview minus 3), has anyone taken or tried taking something from you, by using force or threatening to use force? Did this last happen during the last 12 months, that is, since (month of interview) (year of interview minus 1)?’
2. ‘Apart from the incident(s) just covered, have you in the last 3 years, that is since (month of interview) (year of interview minus 3), been physically attacked? Did this last happen during the last 12 months, that is, since (month of interview) (year of interview minus 1)?’

Responses were recoded into one binary variable; had been exposed to violence (assault or robbery) in the last 1 year vs. not exposed.

Exposure to discrimination was based on responses to a single question: ‘In the past 12 months, have you personally felt discriminated against or harassed on the basis of the following grounds? (1) Ethnic or immigration origin?, (2) Sex?, (3) Sexual orientation?, (4) Age?, (5) Religion or belief?, (6) Disability?, (7) For any other reason?’ Responses to this variable were used to derive a binary variable; exposed to any form of discrimination in the past 12 months vs. not exposed. Violence and discrimination data for both men and women were both collected in a subset of 15 countries. Data were missing for 0.4% of respondents on discrimination and 0.6% of respondents on violence in this subset of countries.

### Ethical review

The survey protocol, which included a Protection Protocol outlining the potential risks during the life cycle of the survey and management strategies to mitigate these, was approved by a relevant national organization (see Supplementary Table). In all countries verbal consent was obtained for each respondent participating and, for children age 15–17 years individually interviewed, adult consent was obtained in advance of the child’s assent. In all countries all respondents were informed of the voluntary nature of participation and the confidentiality and anonymity of information. In addition, in all countries respondents were informed of their right to refuse answering all or specific questions, as well as to stop the interview at any time. UNICEF reviewed the potential uses of MICS data by the applicants before granting access. In light of the above arrangements no further ethical review procedures were deemed necessary.

### Approach to analysis

First, we used simple bivariate descriptive statistics to derive country-level and pooled estimates (with 95% confidence intervals) of the prevalence of disability, happiness and life satisfaction. All prevalence estimates were age-standardised using 5-year age groups against the gender specific 2019 World Standard Population estimates generated by the Institute for Health Metrics and Evaluation (https://ghdx.healthdata.org/record/ihme-data/gbd-2019-population-estimates-1950-2019). Second, we used Spearman’s non-parametric correlation coefficients to estimate the strength of association across countries between PWB and country wealth (pcGNI) and human development potential (HDI). Third, we used mixed effects multilevel Poisson regression to estimate the independent strength of association (adjusted prevalence rate ratios: APRRs) between disability and potential predictors of wellbeing.

Fourth, we used simple bivariate descriptive statistics to estimate country-level prevalence rates for happiness and life satisfaction among women/men with/without disability. We also used Poisson regression to estimate APRRs for women/men with disabilities reporting positive PWB adjusted for between group differences in age (women/men without disabilities being the reference groups). We used two approaches to aggregate data across the 22 countries which had data for both women and men: (1) mixed effects multilevel modelling with random effects specified to allow the intercept and slope for the association between disability and the outcome to vary across countries; (2) restricted maximum likelihood meta-analysis. We also used Spearman’s non-parametric correlation coefficients to estimate the strength of association across countries between absolute and relative inequalities in PWB based on disability status and country wealth (pcGNI) and human development potential (HDI). Within country absolute inequality was defined as PWB of people without disabilities minus the PWB of people with disabilities. Relative inequalities were defined as the APRR reported in Table 3.

Given that choice of cut off for recoding ordinal scales into binary measures may influence results, we undertook several sensitivity analyses using different cut-points to create indicators of life satisfaction and happiness. For life satisfaction, we used the cut-points for current life satisfaction suggested by Gallup [36] to create additional indicators of ‘thriving’ (current life satisfaction 0–6, vs 7–10) and ‘suffering’ (current life satisfaction 0–4, vs 5–10); termed ‘very high’ and ‘moderate’ in our analyses. We created similar indicators for ‘very high’ happiness (somewhat unhappy/very unhappy/neither happy nor unhappy/somewhat happy vs. very happy) and ‘moderate’ happiness (somewhat unhappy/very unhappy vs. neither happy nor unhappy/ somewhat happy/very happy).

Fifth, we used mixed effects multilevel modelling (with random effects specified as above) to examine the extent to which the association between disability and PWB reflected the differing life experiences between people with/without disabilities for the subset of 15 countries for which we had data on exposure to violence and discrimination.

All analyses were undertaken using Stata 16.1 using svy facility to take account of the clustering of observations by country and within country clusters. Mixed effects multilevel modelling of within-country associations was undertaken using the mepoisson command. Meta-analysis was undertaken using restricted maximum likelihood regression. UNICEF’s country-specific women-level weights were used to take account of biases in sampling frames and household and individual level non-response. Given the unequal sample sizes for men and women, all analyses were stratified by gender. Given the small amount of missing data, complete case analyses were undertaken.

## Results

Information (including sample sizes, response rates and the prevalence of disability and PWB indicators) for the 27 surveys is presented in Table 1.

### Prevalence and predictors of disability

Overall, 114.6% (95% CI 114.1–15.2) of women aged 18–49 years were identified as having a disability [9.4% (9.2–9.7) with less severe disability and 4.8% (4.5–5.1) with more severe disability]. Among men, 10.2% (95% CI 8.8–11.8) were identified as having a disability [6.1% (5.2–7.2) with less severe disability and 4.1% (3.5–4.6) with more severe disability]. Multilevel modelling indicated that disability was associated with significantly increased risk of exposure to older age, lower household wealth, lower education, not being currently partnered and violence and discrimination in the past 12 months (Table 2). Country level prevalence of disability was unrelated to either pcGNI (women Spearman’s *r* = − 0.11; men *r* = − 0.09) or HDI (women Spearman’s r = − 0.08; men *r* =  + 0.02).

**Table 2:** Association between disability and potential predictors of PWB

Predictors of PWB	Association with disability (prevalence rate ratio with 95% CI)	
	Women	Men
Older age (35 years or over; reference group 18–34 years	1.60*** (1.52–1.68)	1.34*** (1.24–1.45)
Lower relative household wealth (Quintile 1–2; reference group = quintile 3–5)	1.14*** (1.07–1.22)	1.20*** (1.10–1.30)
Lower educational attainment (Primary or none; reference group = secondary level education)	1.25*** (1.12–1.40)	1.22*** (1.13–1.33)
Urban location (Reference group = rural location)	0.97 (0.92–1.02)	0.91* (0.84–0.98)
Formerly or never married/in union (Reference group = currently married in union)	1.20*** (1.14–1.27)	1.11 ** (1.04–1.19)
Exposed to violence in last year (Reference group = not exposed)	2.09*** (1.78–2.46)	1.58*** (1.37–1.83)
Exposed to discrimination in last year (Reference group = not exposed)	1.93*** (1.83–2.04)	2.04*** (1.48–2.81)

All analyses (with the exception of age) adjusted for between-group differences in age

*p < 0.05, **p < 0.01, ***p < 0.001

### Prevalence and predictors of PWB

Overall prevalence rates among women were 72.2% (95% CI 71.4–72.9) for happiness and 55.5% (95% CI 48.8–56.1) for positive life satisfaction. Prevalence rates among men were 69.5% (95% CI 67.4–71.5) for happiness and 48.0% (95% CI 45.5–50.6) for positive life satisfaction. Country level prevalence of positive life satisfaction was significantly positively related to pcGNI (women *r* =  + 0.79, *p* < 0.001; men *r* =  + 0.75, *p* < 0.001) and to HDI (women *r* =  + 0.75, *p* < 0.001; men *r* =  + 0.69, *p* < 0.001). Country level prevalence of happiness was also significantly positively related to pcGNI (women *r* =  + 0.78, *p* < 0.001; men *r* =  + 0.80, *p* < 0.001) and to HDI (women *r* =  + 0.75, *p* < 0.001; men *r* =  + 0.73, *p* < 0.001) (cf., Graham et al. [37]).

### Disability and PWB

The overall prevalence of happiness was 61.2% (95% CI 60.1–62.4) for women with disabilities, 73.9% (95% CI 73.2–74.6) for women without disabilities, 59.2% (95% CI 56.7–61.7) for men with disabilities and 70.6% (95% CI 68.7–72.5) for men without disabilities.

Country-level estimates are presented in Table 3. Women with disabilities had lower levels of happiness and life satisfaction than women without disabilities in all LMICs. In 23 LMICs these differences were statistically significant. Men with disabilities had lower levels of happiness and life satisfaction in 20 of 22 LMICs, statistically significant in 11. In no country did men with disabilities have significantly greater PWB than men without disabilities. As with overall levels of PWB, both indicators of PWB were significantly and positively correlated with country wealth (pcGNI) and level of human development (HDI) for both men and women with/without disabilities. The strength of the association did not differ significantly by disability status or gender. There were no statistically significant non-parametric correlations between either pcGNI or HDI and the level of absolute or relative disadvantage in PWB experienced by people with disabilities.

**Table 3:** Country-level estimates of the prevalence of positive life satisfaction and happiness among men and women with/without disability

Disability	Women						Men					
	Positive life satisfaction			Happiness			Positive life satisfaction			Happiness		
	Yes	No	APRR	Yes	No	APRR	Yes	No	APRR	Yes	No	APRR
Costa Rica	83.2% (80.5–85.6)	91.0% (89.2–92.5)	0.91*** (0.88–0.95)	79.6% (76.6–82.3)	90.1% (88.6–91.5)	0.88*** (0.84–0.92)	Data not collected					
Montenegro	74.2% (66.5–80.6)	92.9% (89.1–95.4)	0.80*** (0.74–0.87)	76.7% (69.1–82.6)	95.4% (89.9–98.0)	0.82*** (0.77–0.88)	71.2% (49.5–85.0)	90.4% (85.6–93.7)	0.83 (0.66–1.06)	71.0% (56.8–85.8)	97.1% (94.4–98.5)	0.77 (0.58–1.01)
Belarus	76.1% (69.5–82.6)	85.5% (84.0–86.9)	0.85*** (0.78–0.93)	81.3% (75.5–86.2)	92.3% (91.1–93.4)	0.84*** (0.78–0.91)	43.3% (28.7–57.9)	79.2% (76.2–81.9)	0.67* (0.48–0.92)	73.1% (59.9–83.3)	87.0% (84.7–88.9)	0.86 (0.73–1.02)
North Macedonia	53.8% (46.7–60.9)	82.4% (73.0–89.1)	0.63*** (0.59–0.68)	67.0% (59.9–73.8)	90.0% (86.1–93.0)	0.73*** (0.68–0.79)	Data not collected					
Tuvalu	69.5% (65.3–73.4)	76.1% (74.6–77.5)	0.90 (0.71–1.15)	91.0% (90.3–91.7)	96.5% (96.4–96.6)	0.94 (0.76–1.16)	55.8% (51.2–60.4)	60.4% (56.2–64.6)	0.86 (0.54–1.37)	95.6% (91.2–98.1)	91.9% (90.9–92.8)	1.04 (0.73–1.47)
Suriname	73.7% (70.9–76.3)	87.1% (86.1–88.0)	0.88** (0.81–0.95)	73.7% (71.0–76.3)	84.8% (83.8–85.7)	0.88** (0.81–0.95)	76.6% (70.8–81.9)	68.3% (66.4–70.2)	1.04 (0.89–1.23)	77.1% (71.3–82.0)	83.9% (83.9–84.8)	0.91 (0.78–1.06)
Iraq	47.0% (43.2–50.8)	56.0% (54.1–57.8)	0.84*** (0.77–0.91)	56.8% (53.7–59.9)	68.5% (66.8–70.1)	0.83*** (0.79–0.88)	Data not collected					
Georgia	54.5% (50.6–58.5)	72.9% (71.0–74.8)	0.75*** (0.69–0.82)	87.8% (74.3–80.8)	89.9% (88.7–91.0)	0.87*** (0.85–0.93)	49.1% (41.5–57.2)	64.6% (61.5–67.5)	0.65*** (0.53–0.80)	75.2% (64.7–83.2)	85.8% (83.6–87.8)	0.82** (0.73–0.93)
Tonga	82.8% (76.4–87.6)	93.4% (92.1–94.4)	0.90** (0.84–0.97)	86.5% (77.6–92.4)	93.9% (92.5–95.1)	0.91* (0.84–0.99)	88.2% (77.3–94.7)	92.6% (89.7–94.8)	0.93 (0.84–1.02)	88.7% (75.7–95.0)	96.3% (94.7–97.2)	0.92 (0.84–1.02)
Palestine	50.8% (46.2–55.5)	70.7% (68.8–72.6)	0.73*** (0.66–0.80)	69.8% (65.7–73.7)	81.5% (80.3–82.8)	0.87*** (0.82–0.93)	Data not collected					
Samoa	86.0% (80.6–90.1)	90.5% (88.5–92.3)	0.94* (0.90–0.99)	90.1% (85.5–93.4)	92.8% (91.6–93.9)	0.97 (0.93–1.01)	82.9% (71.0–89.8)	91.3% (88.6–93.4)	0.92 (0.83–1.03)	94.5% (84.9–99.1)	94.4% (92.0–96.0)	0.97(0.90–1.05)
Sao Tome & Principe	57.0% (52.0–61.8)	59.5% (56.6–62.4)	0.96 (0.88–1.05)	72.3% (67.9–77.4)	76.3% (73.9–78.5)	0.95 (0.88–1.02)	42.6% (33.0–52.5)	54.1% (50.3–58.0)	0.84 (0.69–1.04)	63.5% (51.7–73.7)	89.2% (86.5–91.6)	0.77** (0.64–0.92)
Mongolia	60.2% (56.5–63.7)	71.2% (69.2–73.0)	0.85*** (0.80–0.90)	73.7% (70.2–77.0)	86.9% (85.4–88.1)	0.86*** (0.82–0.90)	63.2% (57.0–69.0)	64.2% (61.5–66.8)	1.00 (0.91–1.11)	82.5% (77.6–86.6)	86.7% (84.5–88.5)	0.94 (0.89–1.00)
Kiribati	70.8% (66.2–74.9)	77.7% (74.6–80.7)	0.91** (0.85–0.96)	74.3% (69.6–78.4)	83.2% (80.6–85.5)	0.90*** (0.85–0.94)	60.9% (54.0–67.4)	74.8% (70.9–78.4)	0.82** (0.73–0.92)	60.5% (52.9–67.6)	77.3% (73.4–80.8)	0.79*** (0.70–0.88)
Ghana	36.9% (34.0–40.0)	50.4% (48.4–52.4)	0.74*** (0.68–0.80)	63.5% (60.6–66.3)	74.2% (72.4–75.9)	0.85*** (0.81–0.90)	30.2% (25.0–35.9)	42.5% (39.7–45.4)	0.71** (0.59–0.86)	60.9% (53.0–68.3)	75.6% (72.9–78.2)	0.80*** (0.72–0.90)
Zimbabwe	25.7% (22.8–28.9)	36.1% (34.6–37.5)	0.71*** (0.63–0.81)	49.5% (46.2–52.9)	59.1% (57.6–60.7)	0.82*** (0.77–0.88)	22.6% (18.4–27.5)	27.7% (25.8–29.6)	0.85 (0.70–1.04)	54.0% (47.6–60.2)	63.2% (60.9–65.4)	0.85** (0.76–0.96)
Bangladesh	40.5% (39.1–41.9)	49.3% (48.7–49.9)	0.84*** (0.81–0.88)	73.7% (72.4–74.9)	84.5% (84.1–84.9)	0.88*** (0.87–0.90)	Data not collected					
Lesotho	36.6% (32.3–41.2)	46.5% (44.4–48.7)	0.80*** (0.71–0.91)	55.6% (51.1–60.1)	65.6% (63.1–68.0)	0.85*** (0.78–0.93)	28.0% (21.9–35.0)	39.5% (36.2–42.9)	0.73** (0.58–0.92)	66.3% (58.6–73.2)	71.6% (68.3–74.6)	0.93 (0.83–1.04)
Nepal	60.2% (56.2–64.0)	71.6% (69.8–73.0)	0.85*** (0.80–0.91)	47.2% (43.7–50.8)	62.4% (60.6–64.2)	0.73*** (0.68–0.80)	56.0% (50.5–61.5)	67.5% (66.1–68.9)	0.79** (0.67–0.93)	36.1% (30.9–41.6)	64.6% (63.2–66.0)	0.60*** (0.50–0.72)
Guinea-Bissau	69.2% (64.9–73.2)	74.6% (72.2–76.8)	0.93* (0.87–0.99)	88.7% (85.3–91.4)	91.2% (89.9–92.4)	0.97 (0.94–1.01)	49.6% (33.8–66.4)	41.1% (36.6–45.9)	1.12 (0.77–1.62)	85.5% (69.9–95.2)	93.7% (91.3–95.6)	0.86 (0.74–1.00)
The Gambia	41.5% (37.0–46.2)	48.9% (46.9–50.9)	0.85** (0.76–0.95)	64.7% (60.9–68.4)	73.5% (72.2–74.8)	0.88*** (0.83–0.93)	36.9% (31.0–43.2)	50.1% (47.3–53.0)	0.73*** (0.62–0.86)	59.8% (54.1–65.3)	71.6% (68.8–74.3)	0.84*** (0.77–0.92)
Chad	46.3% (43.3–49.4)	47.7% (46.0–49.6)	0.98 (0.92–1.04)	46.1% (43.3–48.9)	56.5% (54.7–58.1)	0.82*** (0.77–0.87)	27.8% (22.2–34.2)	35.8% (32.7–38.9)	0.79* (0.65–0.98)	45.4% (39.6–51.3)	62.7% (59.9–65.5)	0.72*** (0.64–0.82)
Togo	34.9% (31.7–38.3)	44.3% (41.7–46.9)	0.80*** (0.72–0.88)	51.2% (46.9–55.5)	61.0% (59.1–62.9)	0.84*** (0.77–0.92)	30.8% (23.8–38.9)	34.9% (31.1–39.0)	0.87 (0.67–1.11)	53.1% (45.0–61.0)	53.8% (49.9–57.6)	0.99 (0.84–1.16)
DR Congo	30.1% (26.2–34.3)	44.8% (41.1–48.5)	0.68*** (0.60–0.76)	34.1% (29.3–39.2)	51.3% (47.3–55.2)	0.67*** (0.59–0.76)	28.7% (20.8–38.2)	35.3% (30.9–39.9)	0.76 (0.56–1.07)	35.7% (27.8–44.7)	48.7% (44.1–53.3)	0.71** (0.55–0.90)
Sierra Leone	37.0% (33.8–40.3)	47.8% (47.0–48.6)	0.74*** (0.66–0.83)	64.9% (61.6–68.1)	73.5% (72.8–74.2)	0.87** (0.79–0.95)	41.3% (34.7–48.3)	52.9% (51.6–54.1)	0.76* (0.61–0.95)	63.3% (56.3–69.6)	73.7% (72.6–74.8)	0.82* (0.68–0.98)
Central African Republic	39.9% (37.1–42.8)	43.4% (40.7–46.1)	0.93 (0.86–1.00)	39.8% (37.0–42.6)	48.2% (45.6–50.8)	0.83*** (0.76–0.90)	34.0% (27.6–41.1)	42.6% (38.2–47.0)	0.80* (0.66–0.97)	37.7% (31.0–44.9)	43.5% (39.2–47.9)	0.86 (0.72–1.04)
Malawi	35.4% (32.8–38.1)	42.2% (40.8–43.7)	0.91*** (0.86–0.96)	56.1% (53.2–59.0)	62.0% (60.8–63.3)	0.85*** (0.79–0.92)	25.6% (21.0–30.8)	35.1% (32.4–37.9)	0.73** (0.60–0.88)	45.1% (39.4–50.8)	56.7% (54.1–59.2)	0.81*** (0.72–0.91)

*p < 0.05, **p < 0.01, ***p < 0.001

Aggregation of data across countries by multilevel modelling generated pooled age-adjusted prevalence rate ratios for women of 0.85 [(0.83–0.87), *p* < 0.001] for happiness and 0.83 [(0.80–0.87), *p* < 0.001] for life satisfaction, and for men of 0.83 [(0.79–0.88), *p* < 0.001] for happiness and 0.81 [(0.76–0.87), *p* < 0.001] for life satisfaction. Aggregation of data across countries by meta-analysis generated pooled age-adjusted prevalence rate ratios for women of 0.86 [(0.84–0.88), *I*^2^ = 85.9%, *p* < 0.001] for happiness and 0.83 [(0.79–0.87), *I*^2^ = 89.5%, *p* < 0.001] for life satisfaction, and for men of 0.85 [(0.81–0.89), *I*^2^ = 64.2%, *p* < 0.001] for happiness and 0.83 [(0.78–0.88), *I*^2^ = 5.1%, *p* < 0.001] for life satisfaction.

Sensitivity analyses (undertaken using multilevel modelling) generated pooled age-adjusted prevalence rate ratios for women of 0.77 [(0.72–0.81), *p* < 0.001] for ‘very high’ happiness, 0.85 [(0.83–0.88), *p* < 0.001] for ‘moderate’ happiness, 0.79 [(0.75–0.84), *p* < 0.001] for ‘very high’ life satisfaction, and 0.89 [(0.87–0.92), *p* < 0.001] for ‘moderate’ life satisfaction. For men estimates were 0.77 [(0.70–0.83), *p* < 0.001] for ‘very high’ happiness, 0.83 [(0.79–0.88), *p* < 0.001] for ‘moderate’ happiness, 0.84 [(0.77–0.91), *p* < 0.001] for ‘very high’ life satisfaction, and 0.87 [(0.85–0.92), *p* < 0.001] for ‘moderate’ life satisfaction.

Adjusted prevalence rate ratios aggregated across countries by multilevel modelling are presented in Table 4 for the likelihood of higher wellbeing among women and men with more and less severe disabilities (women/men without disabilities being the reference group) in the subset of countries in which data was collected for women/men on exposure to violence and discrimination. In both models (Model 1 adjusted for age, Model 2 also adjusted for relative household wealth, level of education, marital status and urban/rural location and exposure to violence and discrimination), women/men with disabilities had significantly lower wellbeing than women/men without disabilities. Lower wellbeing in Model 2 was independently associated with relative household wealth, not being married/in a union, exposure to violence and discrimination and, to a lesser extent and inconsistently, lower levels of education. Adjustment for differences in living conditions reduced the disability-related inequities in happiness by 24% for women with less severe disabilities, 26% for women with more severe disabilities, 13% for men with less severe disabilities and 12% for men with more severe disabilities Adjustment for differences in living conditions reduced the disability-related inequities in life satisfaction by 22% for women with less severe disabilities, 24% for women with more severe disabilities, 11% for men with less severe disabilities and 22% for men with more severe disabilities.

**Table 4:** Predictors of wellbeing in women and men aged 18–49 years in 15 Countries

	Women				Men			
	Happiness		Positive life satisfaction		Happiness		Positive life satisfaction	
	Model 1	Model 2	Model 1	Model 2	Model 1	Model 2	Model 1	Model 2
Disability status								
No disability	1.00 (reference)	1.00 (reference)	1.00 (reference)	1.00 (reference)	1.00 (reference)	1.00 (reference)	1.00 (reference)	1.00 (reference)
Less severe disability	0.861*** (0.83–0.90)	0.894*** (0.86–0.92)	0.850*** (0.80–0.90)	0.883*** (0.84–0.924)	0.888*** (0.85–0.93)	0.903*** (0.87–0.94)	0.820*** (0.74–0.90)	0.839*** (0.76–0.92)
More severe disability	0.826*** (0.78–0.88)	0.871*** (0.83–0.92)	0.813*** (0.74–0.89)	0.858*** (0.79–0.93	0.788*** (0.71–0.87)	0.814*** (0.74–0.89)	0.839** (0.75–0.94)	0.875* (0.78–0.98)
Age								
18–19	1.00 (reference)	1.00 (reference)	1.00 (reference)	1.00 (reference)	1.00 (reference)	1.00 (reference)	1.00 (reference)	1.00 (reference)
20–24	0.96*** (0.94–0.98)	0.96*** (0.94–0.97)	0.95** (0.92–0.98)	0.95*** (0.92–0.97)	0.96*** (0.93–0.98)	0.95*** (0.93–0.97)	0.87*** (0.82–0.93)	0.87*** (0.83–0.93)
25–29	0.94** (0.90–0.98)	0.93*** (0.90–0.96)	0.94** (0.89–0.98)	0.93** (0.88–0.98)	0.96* (0.94–0.99)	0.94** (0.91–0.98)	0.88** (0.82–0.95)	0.88*** (0.82–0.95)
30–34	0.90*** (0.86–0.95)	0.90*** (0.86–0.94)	0.90** (0.85–0.96)	0.90** (0.85–0.96)	0.94* (0.89–0.99)	0.91*** (0.86–0.95)	0.83*** (0.76–0.92)	0.83*** (0.77–0.90)
35–39	0.90*** (0.85–0.95)	0.89*** (0.86–0.93)	0.91** (0.85–0.96)	0.91** (0.85–0.96)	0.92*** (0.88–0.96)	0.89*** (0.85–0.92)	0.87** (0.79–0.95)	0.87*** (0.81–0.93)
40–44	0.87*** (0.81–0.94)	0.88*** (0.82–0.93)	0.89** (0.83–0.97)	0.90* (0.82–0.98)	0.92** (0.87–0.97)	0.88*** (0.83–0.94)	0.88** (0.81–0.95)	0.88** (0.80–0.96)
45–49	0.89*** (0.84–0.94)	0.90*** (0.85–0.94)	0.91** (0.85–0.98)	0.92* (0.86–0.98)	0.91** (0.86–0.97)	0.88*** (0.83–0.93)	0.86** (0.77–0.95)	0.86** (0.79–0.94)
Household wealth								
Quintile 1 (poorest)		0.84*** (0.79–0.90)		0.83** (0.74–0.93)		0.90*** (0.85–0.95)		0.82*** (0.74–0.90)
Quintile 2		0.88*** (0.83–0.93)		0.84*** (0.77–0.92)		0.90*** (0.87–0.93)		0.82*** (0.76–0.88)
Quintile 3		0.89*** (0.85–0.94)		0.86*** (0.80–0.93)		0.94*** (0.91–0.96)		0.84*** (0.78–0.90)
Quintile 4		0.92*** (0.85–0.94)		0.88*** (0.84–0.93)		0.94*** (0.91–0.97)		0.88*** (0.83–0.94)
Quintile 5 (wealthiest)		1.00 (reference)		1.00 (reference)		1.00 (reference)		1.00 (reference)
Education								
None/pre-primary		0.87 (0.76–1.00)		0.91 (0.79–1.04)		1.03 (0.91–1.17)		1.08 (0.95–1.24)
Primary		0.92* (0.85–1.00)		0.93 (0.85–1.02)		0.97 (0.92–1.01)		0.94** (0.90–0.98)
Secondary or higher		1.00 (reference)		1.00 (reference)		1.00 (reference)		1.00 (reference)
Marital status								
Currently married/ in union		1.00 (reference)		1.00 (reference)		1.00 (reference)		1.00 (reference)
Formerly married/in union		0.80*** (0.74–0.87)		0.79*** (0.76–0.83)		0.80*** (0.74–0.86)		0.83*** (0.75–0.92)
Never married/in union		0.94*** (0.91–0.97)		0.95* (0.91–1.00)		0.95* (0.90–1.00)		1.00 (0.91–1.09)
Urban location^a^		0.99 (0.96–1.02)		1.00 (0.95–1.06)		0.98 (0.95–1.01)		1.01 (0.96–1.07)
Exposure to violence^a^		0.90** (0.84–0.97)		0.90***0.85–0.95		0.95 (0.89–1.02)		0.95 (0.87–1.05)
Exposure to discrimination^a^		0.89*** (0.85–0.92)		0.88*** (0.84–0.93)		0.93** (0.89–0.98)		0.94*** (0.91–0.97)

^a^Reference group = not exposed

*p < 0.05, **p < 0.01, ***p < 0.001

## Discussion

Our analyses of the circumstances of nationally representative samples involving 296,693 women and 66,557 men aged 18–49 years from 27 middle- and low-income countries indicated that women/men with disabilities were less likely than their non-disabled peers to report being happy and to be satisfied with their life. These differences were evident in all countries for women and in 20 of 22 countries for men. Aggregating data across countries indicated that: (1) women with disabilities were 14–15% less likely to be happy and 17% less likely to be satisfied with their lives than non-disabled women; (2) men with disabilities were 15–17% less likely to be happy and 17–19% less likely to be satisfied with their lives than non-disabled men; (3) disability-related inequalities in personal wellbeing were reduced by 22–26% for women and 11–22% for men by adjusting for between-group differences in living conditions and life experiences.

This is, to our knowledge, the first study to report nationally representative estimates for PWB among women and men with/without disabilities in a range of LMICs. The main strengths of the study are: (1) the use of robust nationally representative samples of women/ men aged 18–49 years with high response rates generated by an ongoing survey programme; and (2) the use of alternative analytic approaches to test the robustness of the results. The main weaknesses of the study are: (1) the use of the WGSS to identify people with disabilities; (2) the limited range of indicators of PWB employed; (3) the restricted age-range of participants (18–49 years) and (4) the cross-sectional nature of the data.

The WGSS underestimates the prevalence of disability through its failure to identify people whose disability is associated with functional impairments not included in the WGSS (e.g., mental health related limitations) [22–24]. This may lead to an underestimation of the strength of association between disability and PWB. The second issue means that no conclusions can be drawn about the association between disability in LMICs and other key indicators of PWB (negative affect, eudemonic wellbeing). The third issue means that these results cannot be generalised to older adults (age 50 years and above). The final issue means that no conclusions can be drawn from these data regarding causal links between disability and lower wellbeing. The results are, however, consistent with those from longitudinal studies that suggest that the onset of disability may be associated with subsequent reductions in wellbeing [11, 38].

The results are important on two counts. First, they demonstrate consistent effects of disability-related inequities in PWB across two indicators of PWB, a wide range of LMICs and across women and men. Notably, the differences in PWB between people with/without disabilities were consistent with those reported in high income countries [9, 13] and did not differ significantly within LMICS by either country wealth or indicators of human development potential. Second, they suggest that approximately 25% of the deficit in PWB experienced by people with disabilities may be attributable to differences in living conditions and life experiences in relation to household wealth, level of education, partnership status and exposure to violence and discrimination in the previous year. It is important to note that the living conditions experienced by people with disabilities are not inevitable, but the result of social and cultural practices. As such, equalisation of the living conditions faced by people with/without disabilities through social policy interventions (as required in countries that have signed the UN Convention on Rights of Persons with Disabilities), may have the additional benefit of also reducing inequities in PWB between people with/without disabilities. Finally, the results suggest that future releases of MICS data could prove a valuable resource in monitoring country-level progress to realising Sustainable Development Goal 3 and the extent to which progress is equitable between women and men and between people with/without disabilities.

Future research in this area could focus on three areas: (1) including measures of negative affect and eudemonic wellbeing; (2) examining trends over time is disability related inequities in PWB; (3) better understanding the drivers of disability-related inequities in wellbeing by including a wider range of indicators of differences in living conditions (e.g., loneliness and social isolation, productive employment) and attempting to identify the extent to which these differences mediate the relationship between disability and PWB; and (4) further investigation of differences in the aspirations of people with/without disabilities.

## Supplementary Information

Below is the link to the electronic supplementary material.

Supplementary file1 (DOCX 14 kb)
