# Supplementary material for: The wellbeing of women and men with and without disabilities: evidence from cross-sectional national surveys in 27 low- and middle-income countries
Source: Qual Life Res. 2022 Oct 22;32(2):357–71. doi: 10.1007/s11136-022-03268-y (PMC9911466; doi:10.1007/s11136-022-03268-y)
Supplement: Supplementary file 1 — Supplementary file1 (DOCX 14 kb) [file 11136_2022_3268_MOESM1_ESM.docx]

**Supplementary Table: Ethical Review Arrangements**

| *Country* | *Ethical Review Organization* |
| --- | --- |
| Costa Rica | Instituto Nacional de Estadística y Censos (Costa Rica) |
| Montenegro | UNICEF Country Office Ethical Review Committee (Montenegro) |
| Belarus | Health Media Lab Institutional Review Board (USA) |
| North Macedonia | Health Media Lab Institutional Review Board (USA) |
| Tuvalu | Central Statistics Division (Tuvalu) |
| Suriname | Internal Review Board (Suriname) |
| Iraq | Health Media Lab Institutional Review Board (USA) |
| Georgia | National Centre for Disease Control and Public Health (Georgia) |
| Tonga | Education and Training Review Board (Tonga) |
| Palestine | Health Media Lab Institutional Review Board (USA) |
| Samoa | DHS-MICS Steering Committee (Samoa) |
| Mongolia | National Statistics Office (Mongolia) |
| Kiribati | Social Development Indicator Survey Technical Steering Committee (Kiribati) |
| Ghana | Ghana Statistical Service (Ghana) |
| Sao Tome & Principe | Agência de Protecção de Dados Pessoais (Sao Tome & Principe) |
| Zimbabwe | Ethical Review Board (Zimbabwe) |
| Bangladesh | Technical Committee of the Government of Bangladesh |
| Lesotho | Ethical Review Committee (Lesotho) |
| Nepal | Central Bureau of Statistics (Nepal) |
| Guinea-Bissau | Comité de Pilotagem do MICS6 (Guinea-Bissau) |
| The Gambia | Government and Medical Research Council Scientific Coordinating Committee (The Gambia) |
| Chad | Comité National de Bioéthique du Tchad (Chad) |
| Togo | Comité Consultatif National de Bioéthique (Togo) |
| DR Congo | Comité National d’Éthique de la Santé à travers l’Avis (DR Congo) |
| Sierra Leone | Ethics and Scientific Review Committee (Sierra Leone) |
| Central African Republic | Comité Scientifique de la Faculté des Sciences de la Santé (Central African Republic) |
| Malawi | National Statistical Office (Malawi) |
